# Fostering children’s creativity through LLM-driven storytelling with a social robot

**DOI:** 10.3389/frobt.2024.1457429

**Published:** 2024-12-13

**Authors:** Maha Elgarf, Hanan Salam, Christopher Peters

**Affiliations:** ^1^ Social Machines and Robotics Lab (SMART), Department of Computer Science, New York University in Abu Dhabi (NYUAD), Abu Dhabi, United Arab Emirates; ^2^ Embodied Social Agents Lab (ESAL), Department of Electrical Engineering and Computer Science (EECS), KTH Royal Institute of Technology, Stockholm, Sweden

**Keywords:** social robots, creativity, large language models, conversational artificial intelligence, educational technology, education with children, collaborative storytelling

## Abstract

Creativity is an important skill that is known to plummet in children when they start school education that limits their freedom of expression and their imagination. On the other hand, research has shown that integrating social robots into educational settings has the potential to maximize children’s learning outcomes. Therefore, our aim in this work was to investigate stimulating children’s creativity through child-robot interactions. We fine-tuned a Large Language Model (LLM) to exhibit creative behavior and non-creative behavior in a robot and conducted two studies with children to evaluate the viability of our methods in fostering children’s creativity skills. We evaluated creativity in terms of four metrics: fluency, flexibility, elaboration, and originality. We first conducted a study as a storytelling interaction between a child and a wizard-ed social robot in one of two conditions: creative versus non-creative with 38 children. We investigated whether interacting with a creative social robot will elicit more creativity from children. However, we did not find a significant effect of the robot’s creativity on children’s creative abilities. Second, in an attempt to increase the possibility for the robot to have an impact on children’s creativity and to increase the fluidity of the interaction, we produced two models that allow a social agent to autonomously engage with a human in a storytelling context in a creative manner and a non-creative manner respectively. Finally, we conducted another study to evaluate our models by deploying them on a social robot and evaluating them with 103 children. Our results show that children who interacted with the creative autonomous robot were more creative than children who interacted with the non-creative autonomous robot in terms of the fluency, the flexibility, and the elaboration aspects of creativity. The results highlight the difference in children’s learning performance when inetracting with a robot operated at different autonomy levels (Wizard of Oz versus autonoumous). Furthermore, they emphasize on the impact of designing adequate robot’s behaviors on children’s corresponding learning gains in child-robot interactions.

## 1 Introduction

Advancements in educational tools for children are evolving rapidly. From basic smartphone games Firmansyah et al. (2020) to sophisticated robotic kits like LEGO Mindstorms, Thymio (Riedo et al., 2012), and Cozmo^1^; educational technologies offer a wide spectrum of applications. These tools not only entertain children, but also enhance learning by fostering creativity and programming skills. Among the most advanced and impactful technologies are social robots (Belpaeme and Tanaka, 2021), which facilitate social, emotional, and cognitive development in children (Kozima and Nakagawa, 2006). Integrating social robots into educational settings has the potential to maximize learning outcomes. When used appropriately, robots serve as valuable supporting tools alongside human educators, enhancing the educational experience and fostering a more dynamic and effective learning environment for children. For instance, social robots can help children learn about the physical world through hands-on interactions, such as learning handwriting (Hood et al., 2015; Chandra et al., 2017) or playing basketball (Belpaeme et al., 2018). Moreover, delegating routine educational tasks to robots can free up time for human educators to focus on more complex, creative problem-solving activities. Robots can handle repetitive practice sessions, reducing the likelihood of negative emotional responses from both children and teachers. This results in a more engaging learning experience, as children find robots to be motivating and enjoyable.

Social robots can take up several different roles in a typical encounter with children (Mubin et al., 2013). The robot may act as a teacher that teaches children educational material such as vocabulary Saerbeck et al. (2010) or science (Janssen et al., 2011). Whereas, a robot acting as a learning companion may collaboratively solve educational problems with its human child peer (Hashimoto et al., 2013). Social robots may also aid children’s educational development by acting as novices and allowing children to teach them educational material. For instance, children learned how to improve their handwriting by teaching a novice robot how to write (Hood et al., 2015; Chandra et al., 2017). Varying the robot’s behavior helps to improve children’s learning outcomes. Previous literature suggests that social support (Janssen et al., 2011; Henkemans et al., 2013), eye contact (Huang and Mutlu, 2013) and joint attention (Saerbeck et al., 2010) exhibited by a robot have had a positive impact on children’s learning performance.

Furthermore, social robots that adapt and personalize their behavior to children have been shown to significantly maximize children’s learning performance (Leyzberg et al., 2014); (Schodde et al., 2017). For instance, children responded more positively to a chess-playing robot when it adapted its encouraging behavior to them (Leite et al., 2012). In a storytelling encounter, children were more engaged with the robot, liked both the robot and the story more, and better recalled the story ideas when the robot entrained to their speech (Kory-Westlund and Breazeal, 2019a).

In recent years, researchers in the field of Child-Robot Interaction (cHRI) have been increasingly focusing on the use of social robots to enhance children’s creativity. Creativity is considered a crucial skill for children’s development and an essential asset for their future education and careers (Robinson and Lee, 2011). Moreover, prior research suggests a creativity crisis that occurs when children stop playing and get indulged in a typical educational system that inhibits their imagination and pushes them to be constrained by their surrounding environment and community (Kim, 2011; Csikszentmihalyi, 1997; Torrance, 1968; Torrance, 1966). Research about creativity in cHRI has focused on figural creativity (e.g., drawing or creating visual representations) (Ali et al., 2021a), constructional creativity (e.g., building blocks with LEGO) (Ali, 2019) and verbal creativity (e.g., playing a word game) (Ali et al., 2019). Improving children’s creativity skills through an engaging setting such as storytelling has seldom been investigated in cHRI. Hence, we decided in our work to consider verbal creativity in a collaborative storytelling interaction. We evaluate creativity according to four metrics as per standard practice in previous research (Guilford, 1967; Sternberg, 2005; Torrance, 1966): 1) *fluency of story ideas*: the number of ideas uttered in a story, 2) *flexibility of story ideas*: the variability in story ideas, addressing different topics and categories of story elements, 3) *elaboration of story details*: the amount of details provided in story ideas, and 4) *originality of story ideas*: the element of surprise and novelty in a story idea.

Given that social interactions often lead to mimicry, where individuals adopt behaviors observed in their peers, another form of strong rapport between children and social robots is when children start imitating robots, and thus, learn new skills in an enjoyable way. For example, children who mimic a social robot when the robot models a growth mindset (Park et al., 2017), curiosity (Gordon et al., 2015) and creativity (Ali et al., 2019; Ali et al., 2021b; Ali et al., 2019). Nevertheless, previous work in cHRI on contagion and verbal creativity with children consisted of a simple creativity word game (Ali et al., 2019). Our work aims to address this gap by examining whether children will mimic or be influenced by a robot’s creative actions during a storytelling task. This represents the first investigation of creativity contagion in a storytelling context within cHRI, advancing beyond earlier research that focused solely on verbal creativity through a word game paradigm (Ali et al., 2019).

On the other hand, a key challenge in implementing creativity in a social robot lies in generating behaviors that are not only diverse and original but also contextually appropriate and engaging for specific audiences. Previous work, such as that by Nichols et al. (2020), has demonstrated the potential for fine-tuning large language models (LLMs) to foster creative collaborative interactions. However, the existing methods do not fully address the unique demands of adapting creative behaviors for child audiences, nor do they systematically integrate standard creativity measures such as fluency, flexibility, elaboration, and originality into the fine-tuning process. In our work, we address these gaps by developing fine-tuned LLMs that exhibit different levels of verbal creativity in storytelling interactions with children. Our approach incorporates established creativity measures to guide the generation of verbal responses, allowing the models to produce behaviors that are not only varied and creative but also tailored to the cognitive and emotional needs of young users. This novel adaptation extends the potential of creative AI beyond adult-centered applications, making it suitable for educational and interactive contexts with children.

We aimed at fostering children’s creativity skills through collaborative storytelling interactions with a social robot. Thus, we implemented the following: 1) Study 1: we conducted it as a one-to-one interaction between a child and a social robot in a storytelling encounter. The robot was wizard-ed. 2) The Machine Learning (ML) models: we fine-tuned Open AI GPT-3^2^ Floridi and Chiriatti (2020) to produce 2 ML models that exhibit creative and non-creative behaviors in a collaborative storytelling interaction. 3) Study 2: we deployed our ML models on the same social robot as *Study 1* and conducted a study structured again as a one-to-one storytelling interaction between a child and the robot. The robot was thus autonomous in *Study 2*.

We summarize our contributions in the following points: first, our work is one of the few works that investigate the use of autonomous robots in a collaborative setting with children. Second, we produced two fine-tuned ML models capable of exhibiting different levels of verbal creativity in a storytelling interaction with children^3^. Third, in our work we are investigating whether a robot embedded with creative behavior will stimulate creativity in children (i.e., will children mimic/copy the robot’s behavior and be creative themselves?). Therefore, our work investigates mimicry or contagion in terms of creativity between a robot and a child in a storytelling setting for the first time in cHRI. Fourth, we conducted studies with 141 children at their schools enabling the evaluation of child-robot interactions in real-life scenarios.

The paper is structured in the following manner: Section 2 synthesizes existing research relevant to the study. Section 3 details our methodology, the experimental design and procedures. Section 4 interprets and discusses the findings and limitations. And finally, Section 5 concludes the paper and entails plans for future work.

## 2 Background

In this section we review and synthesize related work in several key areas relevant to the present work including Human-Robot Co-Creativity (HRCC), social robots for collaborative storytelling, and creative artificial intelligence (AI).

### 2.1 Human-robot co-creativity (HRCC)

HRCC is a term coined by Bossema et al. (2023), defined as the creative collaboration between a human and a robot where both exert an effort in response to each other to generate creative outcomes. HRCC has been recently tackled in the field of Human-Robot Interaction (HRI) and cHRI with promising results. The relationship between creativity and collaboration (essentially describing HRCC) in a typical human-robot encounter may take one of two forms: (1) human-robot collaboration for promoting human creativity, and (2) collaborative creativity (Bossema et al., 2023). The first form involves collaboration between a human and a robot that enables both to inspire, motivate and learn from one another. Creativity has long been evaluated as an individualistic trait backed up by autonomy, control, and the ability to generate unique solutions to challenging problems Ali et al. (2021a). Nevertheless, recent research has demonstrated the importance of collaboration and companionship in stimulating human creativity (Kafai, 2012; MacDonald et al., 2000; Miell and MacDonald, 2000; Baas et al., 2008). Therefore, benefits of creative collaboration between a human and a robot include promoting human creativity through an interaction with a creative artificial agent and enriching the field of creative artificial intelligence with human creative data. Examples of this approach comprise human-robot encounters that aim to foster human creativity (Ali et al., 2019; Ali et al., 2021a; Alves-Oliveira et al., 2020a; Alves-Oliveira et al., 2019a; Alves-Oliveira et al., 2020b; Hubbard et al., 2021). Other experiments included building Lego artefacts with a robot (Ali et al., 2021a) and engaging in a drawing activity (Ali et al., 2019; Ali et al., 2021a; Alves-Oliveira et al., 2019b) or a dancing activity (Fabiano et al., 2017) with a robotic agent; all with the aim to stimulate human creativity in children or adults.

The second form is collaborative creativity where both a human and a robotic agent engage together in order to produce a single creative product (Bossema et al., 2023). For instance, in Hinwood et al. (2018), Lin et al. (2020), a user and a robotic agent collaborate together in order to create a shared artwork.

In this paper, we follow the first approach as we present our creative system capable of collaborating and learning from children’s storytelling data in order to generate creative storytelling ideas. We evaluated our robotic system (a social robot) through two user studies in a storytelling encounter with children.

### 2.2 Social robots for collaborative storytelling

Storytelling has long been an activity enjoyed by both children and adults Park (2017). It is essential for human communication and understanding and has been widely adopted as an effective educational tool for aiding children in their social, emotional, and language development and fostering their creativity skills (Kory-Westlund and Breazeal, 2019b; Kory-Westlund and Breazeal, 2019a; Sun et al., 2017; Ali et al., 2021a). Therefore collaborative storytelling activities between humans and social robots has been utilized as a popular activity in HRI. Nichols et al. used a collaborative storytelling system built using the Large Language Model (LLM) Open AI GPT-2 deployed on a Haru robot to create collaborative stories with adults (Nichols et al., 2020; Nichols et al., 2021). Nevertheless, social robots have been more widely used for the purpose of collaborative storytelling with children rather than adults. In Leite et al. (2015), the authors have explored the effects of children engaging in a storytelling interaction with a group of Keepon robots versus one Keepon robot. Results have shown that although in the individual condition children seemed to retain more information from the story, the group condition suggested more positive effects on children’s social skills. Due to the challenges presented by Automatic Speech Recognition (ASR) for children, many studies entailing storytelling encounters between children and social robots have been tele-operated. For instance, researchers have used a wizard-ed robot to investigate its influence on children’s learning performance in terms of language development, as well as the children’s rapport and engagement with the robot (Kory-Westlund and Breazeal, 2019b; Kory-Westlund and Breazeal, 2019a). In another study utilizing a wizard-ed robot, the authors have explored the differences between the insertion of contextual versus non-contextual storytelling ideas into children’s stories (Sun et al., 2017). The results suggested that the contextual condition encouraged children to participate more actively in the activity. Recently, and despite the challenges presented by ASR with children, researchers have been exploring the benefits of using autonomous robots for collaborative storytelling with children. In Zhang et al. (2023), Zhang et al. found that having an autonomous robot guide children through their exploration of a storybook had a more positive impact on the children’s skills than the children’s free exploration of the book without the presence of a robot.

In our work, we used collaborative storytelling between a robot and a child to improve children’s creativity skills. We started by conducting a study with a wizard-ed robot to collect storytelling data with children. We used this data in our second study to develop an autonomous robotic system capable of collaboratively telling stories with children and report on the results of evaluating it.

### 2.3 Creative artificial intelligence (AI)

The term creative Artificial Intelligence (AI) refers to autonomous models that are capable of producing innovative content. Recently, creative AI has been demonstrated in different domains such as fine art by using color segmentation algorithms to produce artificial drawings and paintings (Li et al., 2020) or music by using mechanical operations designed to produce different sounds on musical instruments (Weinberg and Driscoll, 2006a; Weinberg and Driscoll, 2006b). In terms of creative AI in the domain of literature, several attempts have been made to produce artificial poetry and stories. Colton et al. utilized rhyme, word frequency and similarity to produce poetry (Colton et al., 2012). To generate stories, researchers have built hierarchical story models (Fan et al., 2018), created systems that first generate story plots and then develop the story details (Yao et al., 2019) and used fine-tuned LLMs and ranking systems to determine the most convenient story continuations (Radford et al., 2019; Nichols et al., 2020). Another form of creative AI generation is the use of LLMs for educational purposes. LLMs can serve as a way of co-creation of educational material for children Moore et al. (2023), Xiao et al. (2023).

The idea of creative robots as a form of creative AI has also been investigated in the field of HRI where researchers have been developing creative robots and exploring their effects on human users. For instance, Ali et al. found that children who engaged with a creative robot exhibited higher creativity than children who engaged with a non-creative robot in a series of studies comprising verbal, figural and constructional creativity tasks (Ali et al., 2019, Ali et al., 2021a; Ali et al., 2021b). In a different setting, Ayub et al. created and evaluated an assistive robot that learns personalized breakfast options from humans and uses the acquired knowledge to creatively develop new breakfast options (Ayub et al., 2023).

In this work, we fine-tuned an LLM to create two models that aim to enrich the field of creative AI by enabling a robotic agent to creatively and autonomously engage in collaborative storytelling interactions with children. The creative content was generated based on the four standard verbal creativity criteria (Guilford, 1967; Sternberg, 2005; Torrance, 1966): fluency, flexibility, elaboration and originality.

## 3 Methodology

In this section, first, we present our research questions and hypotheses. Then, we detail the two studies that we conducted and the models that we implemented to promote children’s creativity.

### 3.1 Research questions and hypotheses

Our aim in this research is to foster children’s creativity through child-robot collaborations. Therefore, we posit the following research questions:

RQ1: Does interacting with a creative robot stimulate creativity in children in a collaborative storytelling interaction?

To answer this research question, we conducted *Study 1: Once upon a story*, structured in two conditions: creative versus non-creative. In both conditions, the robot engaged with a child in a one-to-one interaction where they were collaboratively telling a story together. In the creative condition, the robot was generating creative ideas. In the non-creative condition, the robot was using less creative ideas to add to the collaborative story. Due to speech recognition challenges with children, the robot was wizard-ed. Moreover, the tele-operator controlling the robot was chosen from a pool of creative versus non-creative ideas to add to the story with respect to the study condition. Therefore, in most instances, the robot’s addition to the story was rather non-contextual (i.e., was not relevant to the child’s previous story idea).

According to previous research, children who engaged with a creative robot were more creative than children who engaged with a non-creative robot in creative tasks of verbal, figural and constructional nature (Ali et al., 2019; Ali et al., 2021a; Ali et al., 2021b). We investigated the same concept in *Study 1* but in a storytelling context for the first time in cHRI and hence, we formulated the following first hypothesis:

H1: Children who interact with the robot in the creative condition will exhibit higher creativity in their storytelling ideas than children who interact with the robot in the non-creative condition.
RQ2: Does interacting with a creative autonomous robot using contextual story ideas stimulate creativity in children in a collaborative storytelling interaction?

The results of *Study 1* were not significant as explained in detail in Section 3.2.5. According to prior research, using contextual story ideas helped children speak more and made the storytelling activity less challenging for children than using non-contextual story ideas (Sun et al., 2017). Thus, we questioned whether altering the robot’s behavior to contextual autonomous behavior while collaborating with the child in order to tell a story will smooth the flow of the interaction and result in more creative behavior from the children in the creative condition. We developed two autonomous models capable of collaboratively engaging in storytelling interactions with human users and generating contextual content. One model generated creative story ideas and the other generated non-creative story ideas. We deployed our models on a social robot and evaluated them in *Study 2: CreativeBot*; also structured in creative and non-creative conditions. We hypothesized that:

H2: Children who interact with the autonomous robot using contextual ideas in the creative condition will exhibit higher creativity in their storytelling ideas than children who interact with the autonomous robot using contextual ideas in the non-creative condition.
RQ3: Does the collaborative nature of the interaction have an impact on children’s expressed creativity? If yes, what is it?

As a reflection on *Study 1*, we questioned the effect of the collaborative nature of the interaction on children’s creativity skills and children’s perception of the robot. We wanted to explore whether alteration of the nature of the activity (collaborative versus non-collaborative) will yield different results. In our *Study 2*, in addition to structuring the encounters in creative versus non-creative conditions; we further divided them into collaborative versus non-collaborative groups. Hence, we had 4 study conditions: creative collaborative, creative non-collaborative, non-creative collaborative and non-creative non-collaborative. As per Kafai (1995), Ali et al. (2019), Ali et al. (2021a), collaboration stimulates more creativity from children. Hence, our hypothesis with regard to the third research question stated that:

H3: Children who interacted with the robot in the collaborative conditions will exhibit higher creativity skills than children who interacted with the robot in the non-collaborative conditions.

### 3.2 Experiment 1: once upon a story

#### 3.2.1 Participants

The experiment took place at the local Museum of Technology in Stockholm, Sweden, where 38 children participants were recruited, aged 5–10 years old ( Mean=7.84 , SD=1.61 ). Six children who either withdrew from the activity or did not interact with the robot were excluded, leaving data from 32 participants (15 males, 17 females) for analysis.

#### 3.2.2 Apparatus and stimuli

##### 3.2.2.1 Creativity pre-test

Before starting the experiment, we asked each child to solve a standard creativity assessment pre-test [explained in Kahn Jr et al. (2005)] to avoid any biases stemming from the original child’s creativity level. We opted to split our sample into two evenly matched groups based on creativity levels, age, and gender and allocated them to either the creative or the non-creative condition.

##### 3.2.2.2 Experimental design

**FIGURE 1:**
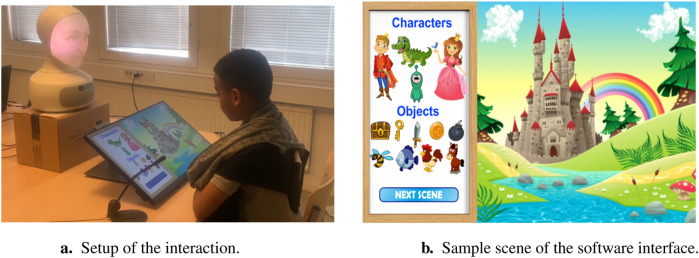
Experimental design of *Study 1* and *Study 2*. **(A)** The study setup used in both experiment 1: once upon a story and experiment 2: creativeBot. **(B)** The castle-themed software interface used in both studies.

We designed the encounter between the child and a tele-operated Furhat robot as a one-to-one interaction and we used the Swedish language. We asked the child to engage in a collaborative storytelling game by alternating with the robot and uttering ideas one by one about their shared story. For simplicity and consistency, we implemented a software interface to aid the child in generating ideas. The software interface had a castle theme, several scenes, 4 characters, and 9 objects to choose from in order to tell the story. Figure 1 displays the setup of the interaction and the details of the storytelling software.

**FIGURE 2:**
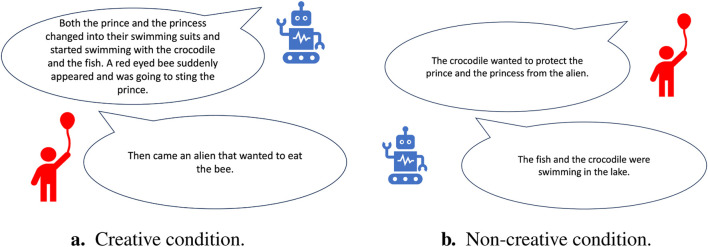
Sample generated story ideas in experiment 1: once upon a story. **(A)** In the creative condition, the robot used creative ideas. **(B)** In the non-creative condition, the robot used non-creative ideas.

To generate the behavior of the robot, the tele-operator randomly chose from a pool of ideas that was extracted from a storytelling dataset previously collected with children who used the same software (Elgarf et al., 2022a; Elgarf et al., 2021a). The tele-operator selected the ideas labeled as creative versus non-creative according to the corresponding study condition. The ideas were labeled according to their originality through an online survey that was administered before the study. 100 survey participants rated each idea on a scale from 1 (extremely non-original) to 5 (extremely original). Ideas rated higher than 3 were classified as creative, while ideas rated lower than 3 were classified as non-creative. The robot in the creative condition generated more ideas (fluency aspect of creativity) and used ideas that were rated as more original (originality aspect of creativity). In contrast, the robot in the non-creative condition generated less ideas that were also rated as less original. Examples of story ideas generated by both the robot and the child in the storytelling encounter in both conditions are presented on Figure 2. For more details about the design of the study and the story ideas used, please refer to Elgarf et al. (2021b).

##### 3.2.2.3 Setup

We conducted the experiment in an isolated separate room of the museum. We positioned two cameras in the room to capture both frontal and profile views of the interaction. Moreover, we installed a microphone beneath the screen to ensure high-quality audio recordings. The child was seated facing the robot, with a touch screen displaying the software interface placed on the table separating them. The child could navigate between the different scenes and move characters and objects all around the screen to tell the story with the robot.

#### 3.2.3 Procedures

The study procedures were approved by the local institution’s ethical committee. On the day of the study, before the interaction started, an experimenter collected demographic information about the children from their parents and provided consent forms for parental approval. The same experimenter managed all logistical aspects, including collecting questionnaires, assigning participant IDs, and managing video and audio data recording. Parents of the children were permitted to observe the experiment from a distance within the room without providing assistance. Another facilitator proficient in the Swedish language welcomed the children, guided them through the experiment, and remotely controlled the robot. Children were first asked to start with the pre-test and then interact with the robot. They were informed that they will engage in a collaborative storytelling game with the robot and that it will suggest ideas to add to their story. They were asked to contribute their own thoughts as well. To ensure consistency in evaluating creativity measures across different participants, the storytelling activity was capped at a maximum of 10 min. Following this, the children completed a brief questionnaire regarding the robot and the game. The experiment typically lasted between 15 and 20 min per child.

#### 3.2.4 Measures

We evaluated our hypotheses by measuring the metrics illustrated in Table 1 from the children stories throughout the storytelling game. To evaluate our data, we used footage from frontal cameras, resorting to lateral videos only when frontal footage was missing or incomplete. Initially, a native speaker transcribed the video data and created English subtitles to facilitate coding by English speakers. Then we assessed children’s fluency and originality of ideas through behavioral coding analysis using the ELAN software^4^ (Wittenburg et al., 2006). We developed a coding scheme following the guidelines outlined in Ongena and Dijkstra (2006). Then, both a primary and a secondary coder annotated 25% of the videos selected randomly to assess inter-rater reliability, following standard practices (Chorney et al., 2015). Our analysis yielded a Cohen’s Kappa value of 0.85 denoting a high agreement between coders as per behavioral psychology research (Watkins and Pacheco, 2000). Therefore, the primary coder proceeded with annotating the rest of the data idependently.

**TABLE 1:** Objective measures assessed for experiment 1: once upon a story.

Metric	Definition
Fluency of story ideas	Assessed as the number of ideas each child generated during the storytelling game
Originality of story ideas	Evaluated based on the children’s use of unexpected and uncommon ideas. Each idea was rated on a pre-defined scale from 1 to 3 for originality. The final originality score was determined by calculating the weighted average of the originality ratings for all ideas generated by the child during the story

We further used a post-interaction questionnaire administered as a modified, simplified version of the Godspeed questionnaire (Bartneck et al., 2009) to evaluate three aspects: the appeal of the robot to the children, their perceived intelligence of the robot and their enjoyment of the storytelling game.

#### 3.2.5 Results

**FIGURE 3:**
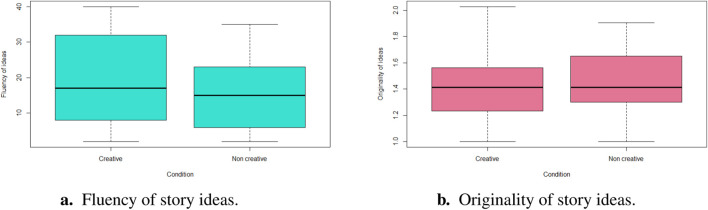
Statistical analysis of experiment 1: once upon a story. There was no significant effect of the robot’s creativity condition on children’s fluency or originality of story ideas. **(A)** Fluency of story ideas. **(B)** Originality of story ideas.

We performed our statistical analysis using a Wilcoxon signed-rank non-parametric test since our data was non-normally distributed. We defined the condition (creative versus non-creative) as our independent variable. We found no significant differences for both creativity measures: fluency (p=0.46,Mean=17.28,SD=11.28) as displayed on Figure 3A and originality of children’s ideas (p=0.67,Mean=1.44,SD=0.27) as presented on Figure 3B between the creative and non-creative conditions.

Furthermore, for children’s perceptions of the robot, the data followed a normal distribution. Therefore, we administered a one way MANOVA parametric test using the condition (creative versus non-creative) as the independent variable. Results have confirmed that no significant effect of the robot’s condition was observed on children’s likeability of the storytelling game (p=0.17,Mean=4.06,SD=0.76) , children’s likeability of the robot (p=0.47,Mean=4.4,SD=0.71) as well as their perceived intelligence of the robot (p=0.26,Mean=3.81,SD=0.93) .

We noticed that during the interaction, the robot’s story ideas sounded in many cases non-contextual and irrelevant to the children’s stories. The robot was controlled by a human who chose story ideas randomly from a limited pool of ideas. Consequently, children were frustrated at the robot for its non-contextual interference to their stories. We therefore decided to conduct *Study 2* in the same setting but with an autonomous instead of a tele-operated social robot to render the interaction more coherent. The robot listens to the child’s ideas, understands them and generates a relevant story continuation. We generated creative and a non-creative ML models (see Section 3.3) to be deployed on a social robot to render it autonomous and be able to use it in both creative and non-creative conditions. Furthermore, we were questioning whether the collaborative nature of the interaction had an impact on the results. Thus, we added a couple of conditions in *Study 2* (explained in detail in Section 3.4) that allowed us to explore the impact of the collaborative aspect of the interaction on children’s creativity skills.

### 3.3 The creative and non-creative ML models

To generate the creativity aspect of the robot’s behaviour, we fine-tuned Open AI GPT-3^2^ (Floridi and Chiriatti, 2020) to produce a creative model and a non-creative model. We used training data provided from a previously collected dataset Elgarf et al. (2021a), Elgarf et al. (2021b) of child-robot interactions in a storytelling context using the same storytelling software that we are using in this research. We extracted children’s story ideas from the dataset and labeled them as creative versus non-creative ideas according to a creativity score. We used the four creativity measures (fluency, flexibility, elaboration and originality) explained in Section 1 to calculate the creativity score as a weighted average of the four metrics for each idea. We evaluated each creativity metric as described in Table 2. For the fluency, flexibility and elaboration we defined a coding scheme to assign corresponding scores of the three variables to each statement. Two coders double-coded 10% of the data as suggested by previous research Chorney et al. (2015). The coding scheme was demonstrated to be valid and clear by their agreement score of 87.12%. Hence, the primary coder coded the rest of the data independently.

**TABLE 2:** Creativity measures used for labeling training data to generate creative behavior from our ML models.

Metric	Definition
Fluency of story ideas	Calculated as the number of story elements used by the child in a given sentence
Flexibility of story ideas	Measured as the number of different story element categories addressed in a given statement (e.g., action, time, location … etc.)
Elaboration of story details	Assessed as the total number of words in a given sentence to get an approximation of the amount of details provided in each story idea
Originality of story ideas	Measured through an online survey answered by 106 participants that rated each statement according to its originality on a scale from 1 to 5. We then calculated the originality score as the average originality rating for each story idea

We then validated our models through an online survey with 26 users. The survey was comprised of 10 stories; 5 stories generated by each the creative and non-creative models. Users were asked to rate each story on a scale from 1 (extremely non-creative) to 5 (extremely creative). We analyzed our data by applying a one-way ANOVA parametric test using the creativity level as an independent variable (creative vs non-creative). Results confirmed the functionality of our models. Users rated the creative model as significantly more creative than the non-creative model (Creative:Mean=3.36,SD=0.98,Non−creative:Mean=2.84,SD=1.1,p<0.001) . For more details about the training and assessment process of our ML models, please refer to Elgarf and Peters (2022).

### 3.4 Experiment 2: creativebot

#### 3.4.1 Participants

Our second experiment was conducted in English language. We therefore recruited 103 children (Male = 54, Female = 49) from 3 British international schools in Stockholm, Sweden (aged 7–9 years old, Mean=7.88,SD=0.77 ). Due to either missing data or inconsistencies in data collection, data from 10 users was excluded. We structured our experiment into four conditions: creative collaborative (24 users), creative non-collaborative (23 users), non-creative collaborative (23 users) and non-creative non-collaborative (23 users).

#### 3.4.2 Apparatus and stimuli

##### 3.4.2.1 Creativity pre-test

To avoid bias resulting from children’s natural level of creativity, we administered the pre-test -outlined in Kahn Jr et al. (2005)- to assess children’s creativity skills prior to the storytelling interaction. We therefore ensured that the 4 experimental groups were balanced in terms of children’s creative abilities. We also confirmed that the 4 groups were balanced in terms of number of participants in the group, and children’s age and gender.

##### 3.4.2.2 Experimental design

**FIGURE 4:**
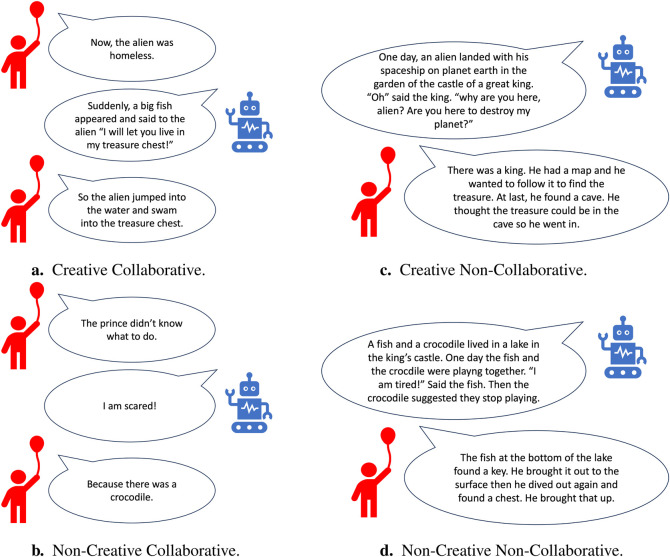
Snippets of some stories produced in the different conditions in experiment 2: creativeBot. In the collaborative conditions **(A, B)** the child and the robot took turns and collaborated together in order to tell a story. In the non-collaborative conditions **(C, D)** the robot told a story to the child first and then asked the child to tell it a story. In the creative conditions **(A, C)** the robot used creative content while in the non-creative conditions **(B, D)** the robot used non-creative content.

Similarly to our previous study, we designed this experiment as a one-to-one interaction between a child and a Furhat robot. Both the robot and the child engaged together in a collaborative storytelling game mediated by the same storytelling software used in the previous study as shown on Figure 1. However, contrary to the previous study, the behaviour of the robot was autonomous rather than wizard-ed. Furthermore, instead of having only 2 conditions (creative versus non-creative), we added a couple of conditions to investigate the effects of the collaborative aspect of the encounter. In the collaborative conditions, both the robot and the child alternated by telling ideas one by one to add to the story. Whereas, in the non-collaborative conditions, the robot first told a story to the child and then asked the child to tell it another story. Table 3 summarizes the four robot’s conditions implemented in *Study 2*. Furthermore, in Figure 4, we present sample story ideas generated in the four different experimental conditions.

**TABLE 3:** Experimental conditions for experiment 2: creativeBot.

Experimental condition	Explanation
Creative Collaborative (CC)	The robot and the child collaborated together in order to tell the story. The robot was exhibiting creative behaviour during the interaction
Creative Non-Collaborative (CNC)	The robot first told the child a story and then asked the child to tell it a story back. The robot exhibited creative behaviour while telling the story
Non-creative Collaborative (NCC)	The robot and the child collaborated together in order to tell the story. The robot exhibited non-creative behaviour during the interaction
Non-creative Non-Collaborative (NCNC)	The robot told a story to the child and then asked the child to tell it a story back. The robot was exhibiting non-creative behaviour during the encounter

Prior research has discussed limitations for the use of social robots with children. Speech recognition is regarded as the most challenging aspect of child-robot interactions. In our collaborative conditions, the robot listened to the child’s input, understood it and generated an idea to continue the story. Hence, to overcome speech recognition errors, we implemented the robot’s behavior in a way that allows it to understand the full context and ignore irrelevant minor details. On Table 4, we show an example of how the robot handled a couple of speech errors that occurred. The robot managed to preserve the context and respond in a way that prevented confusing the child. For more details about the design of the study and the robot’s cognitive behavior, please refer to Elgarf et al. (2022b).

**TABLE 4:** Examples of errors in speech recognition and how the robot handled them.

Child’s input	What the robot heard	The robot’s response
But the prince had a pet bee which helped him	But the prince had a pet fee with Stelton	The alien was scared! -the alien was initially going to attack the prince.-
Then the alien came from outer space	Ben 10 alien came from outer space	The alien wanted to kill the prince with the sword

##### 3.4.2.3 Setup

The study took place in a quiet room on the school premises where the child and the robot were seated face-to-face with the touch screen displaying the software interface between them. In the collaborative conditions, children were able to navigate the characters and objects around the screen to tell the story with the robot. In the non-collaborative conditions, the children watched a video representation of the same software where characters and objects were moving around the scene according to the robot’s speech. We collected demographic data as well as audio and both lateral and frontal video recordings.

#### 3.4.3 Procedures

The study procedures were approved by the local institution’s ethical committee. A couple of weeks before the study, consent forms allowing the children’s participation in the study, the data collection and the publishing of the data were sent to the children’s parents for their signature. On the day of the study, the experimenter greeted the children and then proceeded to conduct the experiment. She first asked the children to complete the creativity pre-test and then interact with the social robot. The robot guided the interaction according to the respective condition. The interaction was open-ended, allowing the child to end it at any time by saying “the end”. The interaction duration varied between 5 and 25 min. Finally, the interaction concluded by the child responding to a brief questionnaire regarding the robot and the game.

#### 3.4.4 Measures

To assess our hypotheses, we transcribed the audio files, or the video files whenever the audio was not available. We developed a coding scheme to assess expressed creativity in children’s stories as per the guidelines in Ongena and Dijkstra (2006). To validate the coding scheme, 20% of our data was doubled-coded using the ELAN software^4^ (Wittenburg et al., 2006) by two coders as per standard practice (Chorney et al., 2015). The two coders had an inter-rater agreement of 89.5% . The rest of the data was then equally and randomly assigned to the two coders to finalize the behavioural coding analysis independently. Our coding scheme measured the metrics explained in Table 5.

**TABLE 5:** Objective measures assessed for experiment 2: creativeBot.

Metric	Definition
Fluency of story ideas	Calculated as the number of elements that the child uttered in their stories
Flexibility of story ideas	Calculated as the number of different categories (e.g., action, time, location … etc.) that the child’s story elements belonged to
Elaboration of story details	Calculated as the difference between the number of words uttered by the child and the number of story elements she included in her stories
Originality of story ideas	Evaluated as the element of surprise and novelty in children’s ideas. Each story idea was rated on a scale from 1 to 3 for originality. The final originality score for each child was calculated as the average originality of all her story ideas

Moreover, we modified the Godspeed questionnaire (Bartneck et al., 2009) to evaluate subjective measures that entailed the children’s likeability of the game, likeability of the robot, likeability of the robot’s ideas and their perception of the robot’s intelligence.

#### 3.4.5 Results

To analyze our results, we investigated three aspects: effects of the robot’s creative behavior on children’s creativity skills, effects of the collaborative nature of the interaction on children’s creativity skills and children’s perceptions of the robot in the creative versus non-creative conditions and in the collaborative versus non-collaborative conditions. Results were as follows:

##### 3.4.5.1 Effects of the robot’s creative behavior on children’s creativity skills

**FIGURE 5:**
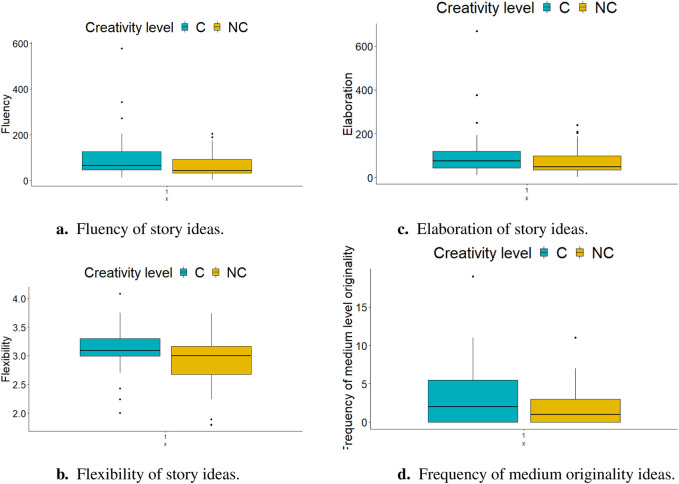
Statistical analysis of experiment 2: creativebot. Children were significantly more fluent, more flexible and more elaborate in their story ideas in the creative conditions than in the non-creative conditions **(A–C)** respectively.) **(D)** Children used more medium originality level ideas in the creative conditions than in the non-creative conditions.

Our sample followed a non-normal distribution. Thus, as per standard practice, we applied a log transformation Lee (2004) to normalize the data and ran a MANOVA parametric test and found the following effects on the different children’s creativity metrics:

• Fluency: children assigned to the creative conditions significantly exhibited higher fluency than children assigned to the non-creative conditions (p<0.01,Mean=83.68,SD=79.67) as displayed on Figure 5A.
• Flexibility: children in the creative conditions significantly expressed higher flexibility than children in the non-creative conditions (p<0.05,Mean=3.02,SD=0.41) as presented on Figure 5B.
• Elaboration: children assigned to the creative conditions were significantly more elaborate than children assigned to the non-creative conditions (p<0.05,Mean=88.45,SD=87.7) as shown on Figure 5C.
• Originality: there was no significant effect of the robot’s creativity level on children’s average level of originality. However, children in the creative conditions significantly expressed more medium originality level ideas (level 2 on a scale from 1 to 3) than children in the non-creative conditions (p<0.05,Mean=2.68,SD=3.27) as displayed on Figure 5D.

##### 3.4.5.2 Effects of the collaborative nature of the interaction on children’s creativity skills

We administered a MANOVA parametric test to measure the effects of the collaborative nature of the interaction on children’s creativity skills. We found no significant effect of the type of interaction (collaborative versus non-collaborative) on the four creativity metrics in children’s stories (fluency, flexibility, elaboration and originality).

##### 3.4.5.3 Children’s perceptions of the robot in the creative versus non-creative conditions and in the collaborative versus non-collaborative conditions

Our sample followed a non-normal distribution. Applying a log transformation did not yield to normally distributed data and thus, we conducted a couple of Wilcoxon signed-rank non-parametric tests.

First, we investigated the effects of the creativity level of the robot on children’s perceptions of the robot. We found that children in the creative conditions perceived the robot as significantly smarter than in the non-creative conditions (p<0.05,Mean=4.38,SD=0.89) . Furthermore, children significantly liked the robot’s story ideas more in the creative conditions than in the non-creative conditions (p<0.05,Mean=4.27,SD=0.92) . We found no significant effects of the robot’s creativity level on the likeability of the robot and the likeability of the storytelling game.

Second, we evaluated the effects of the collaborative nature of the interaction on children’s perceptions of the robot. Children liked the game (p<0.05,Mean=4.27,SD=0.82) and the robot’s story ideas (p<0.05,Mean=4.27,SD=0.92) more in the non-collaborative conditions than in the collaborative conditions. We found no significant effects of the collaborative nature of the interaction on children’s likeability or perceived intelligence of the robot.

## 4 Discussion and limitations

In our *Study 1*, we investigated whether a wizard-ed robot portraying verbal creativity in a collaborative storytelling interaction will render children more creative in their story ideas. Despite previous research that suggests that interacting with a creative robot in a different setting of figural, constructional and verbal creativity yields to more creative behavior from children Ali et al. (2019), Ali et al. (2021a), Ali et al. (2020b); in our study, results showed no significant effect of changing the robot’s behavior between creative and non-creative on children’s creativity skills. Thus, **H1** was rejected. We attribute the results to the fact that the tele-operator wizarding the robot chose story ideas randomly from a limited creative versus non-creative pool of ideas which led to the children expressing their frustration at the robot’s non-contextual changes to their story lines. Figure 2B displays an example of the robot’s non-contextual addition to a child’s story where the robot uses the crocodile to generate a new story idea without building on the child’s previous idea.

We also questioned whether the collaborative nature of the interaction had any effect on the results. Therefore, we designed a second study to investigate: 1) whether changing the robot’s behavior from non-contextual to contextual by rendering the robot autonomous will affect our results. The robot will listen to the child’s idea and then will add another idea to complete the story; 2) whether structuring our conditions in collaborative versus non-collaborative settings will have an effect on the children’s creativity performance and their rapport with the robot.

We modified the robot’s behavior from wizard-ed non-contextual behavior in *Study 1* to autonomous contextual behavior in *Study 2*. In a previous study, children were more interactive with a robot that provided contextual story ideas in a storytelling game than with a robot that provided non-contextual ideas Sun et al. (2017). So as hypothesized, the change led to significant results in terms of children’s creativity skills. Children who interacted with the robot in the creative conditions exhibited higher creativity in measures of fluency, flexibility, elaboration and frequency of the ideas of medium originality level, than children who interacted with the robot in the non-creative conditions. However, there was no effect of the robot’s creativity level on the average originality expressed by children in their stories. An aspect we used in our coding scheme to rate the highest level of originality is when children mixed both elements in the software and elements from their imagination to create their stories. As a comparison shown on Figures 4C, D, the child in the non-creative non-collaborative condition used merely elements present in the storytelling interface (fish, lake, key, chest). However, the child in the creative non-collaborative condition used both elements in the software (king, treasure) and created other elements that are not displayed in the software (cave, map); suggesting higher originality expressed by the child in the creative condition. Nevertheless, by analyzing all the children’s data, the difference in the average originality in children’s stories between the different conditions was insignificant. Hence, **H2** was partially supported. These results suggest that a robot that portrays creative behavior helps children be more fluent in terms of their number of ideas, use ideas related to different topics and incorporate more details while telling a story. However, they do not generate original ideas despite the robot being original in its story generation during the interaction.

Results further shed light on the variability of outcomes stemming merely from changes in the robot’s autonomy level while being used in the exact same setting. It suggests the lack of generalization of studies conducted with wizard-ed robots to real-life scenarios with autonomous robots. Nevertheless, these results contradict the results of a previous study that found that there was no significant difference in user enjoyment and users’ ratings of the robot’s response time when interacting with an autonomous versus a teleo-perated robot (Tozadore et al., 2017). Hence, more research is needed to explore this aspect.

Contrary to our hypothesis, there was no significant effect of the collaborative nature of the interaction on children’s creativity skills. **H3** was therefore rejected. According to prior research, collaboration fosters creativity (Kafai, 1995; Ali et al., 2019; Ali et al., 2021a). Therefore, we attribute our results to the type of interaction (collaborative versus non-collaborative). In the future, structuring our study into collaborative versus competitive may yield different results. Furthermore, children liked the game and the robot’s story ideas more in the non-collaborative conditions than in the collaborative conditions. A possible explanation is that even with the improvement of the robot’s contextual behavior in the collaborative conditions; children were frustrated at the robot’s interference with their story lines and wanted more independence and freedom in their own stories. Nevertheless, in the non-collaborative conditions, the robot told a story and then asked the child to tell it a story back, thus giving her a chance to freely express herself and unleash her imagination and more importantly making her feel listened to.

An interesting and relevant aspect in our studies is the role of the robot’s used language in the children’s creative processes. Different languages use different ways to denote the same concepts. For example, if we want to use the word *grandmother* in Swedish language we need to specify if it is the maternal or paternal grandmother because they are denoted with different words. This implies that in the Swedish language, using the word *grandmother* offers more details about the family relationship between two people than using the same word in English. Scholars have long been intrigued by how languages shape thoughts and impact different cognitive skills Boroditsky (2011). Previous research suggests that bilingualism is positively correlated with creativity only when the two languages are in two different cultural contexts (for example, a person speaking both Spanish and English rather than Spanish and Galician) de Prada Creo et al. (2023). We therefore question whether language had an impact on children’s creativity skills in both our studies. In *Study 1*, children interacted with the robot in Swedish language and were all native Swedish speakers. *Study 2* was conducted in a British international school in Sweden where students were expats speaking fluent English. We did not collect information pertaining to whether English was their first language and whether they were fluent in another language. We addressed the language discrepancy between both studies by comparing children in each study to their counterparts in the same group: children in the British international school encountering the creative robot were compared to children in the British international school encountering the non-creative robot, both interacting with the robot in English. Children’s demonstrated high levels of creativity may have been impacted by the children’s multilingualism which calls for more research about the use of social robots for language and creativity development simultaneously for children.

Our aim with this work was to foster children’s creativity through a storytelling interaction with a social robot. We evaluated children’s creativity during the interaction and hence, we have no guaranteed evidence about the generalization of the increased creativity performance in real-life situations. In longer term interactions, it is unclear whether similar results should be expected due to the novelty effect of the robot wearing off.

Another limitation was in our second study where children interacted with an autonomous robot. The autonomous behavior of the robot was limited to the storytelling part. As soon as both the robot and the child started the storytelling game, the robot perceived any words that the child uttered as a part of the story and therefore tried to build on it, resulting in some confusion from the child. However, there were very few instances of this occurring throughout the study.

Finally, there was a lack of consistency of the definitions and number of creativity variables measured in both studies. However, we explain this by the consistency in definitions within the study itself. The creativity variables that were used to generate the robot’s creative behavior in each study were the same variables used to assess the creativity in children’s stories in the same study. This evaluation strategy is thus aligned with our purpose of investigating whether children will mimic a robot’s creative behavior.

## 5 Conclusion and future work

In this work, we fine-tuned Open AI GPT-3 to generate 2 ML models to produce different levels of collaborative storytelling behavior in terms of creativity, deployed the models on a social robot and conducted two studies that investigated the effects of interacting with a creative robot on children’s creativity skills. We used different modes of operation for the robot in the two studies: in the first study, the robot was wizard-ed and in the second study the robot was autonomous. We found no significant effect of the robot’s creative behavior on children’s creativity skills or children’s perceptions of likeability of the robot when the robot was wizard-ed. Nevertheless, results were promising when the behavior of the robot was automated, suggesting that interacting with a creative robot helps children express higher creativity in a storytelling game. Children who interacted with the autonomous creative robot perceived the robot as significantly smarter than children who interacted with the autonomous non-creative robot. Furthermore, we investigated the effects of collaboration on children’s creativity skills by structuring our second study in a collaborative versus non-collaborative nature. However, we found no significant effect of the collaborative nature of the interaction on children’s creativity skills.

In the future, we plan to conduct longitudinal studies to measure the effects of interacting with a creative robot on children’s creativity development on the long run. Furthermore, implementing the robot as a fully autonomous agent capable of differentiating between children’s story ideas and random irrelevant comments will render the interaction more fluid and realistic. It may even lead to better results in terms of children’s creativity skills and children’s perceived intelligence of the robot. A better strategy in the future to infer children’s likeability of the robot and their engagement with it is by using automatic engagement detection (Salam et al., 2023). Personalization is another interesting future direction. For instance, we can infer children’s engagement with the robot and personalize its social and creative behaviors to each child to maximize their learning gain as suggested in Chithrra Raghuram et al. (2022).

Finally, due to the social nature of storytelling interactions, we also plan to incorporate more social cues in the future in the robot’s behavior such as facial expressions or head movements to investigate their effects on the flow of interaction as well as children’s creativity and social development. Instead of evaluating the system in a collaborative versus non-collaborative setting that had no significant effect on children’s creativity skills, we plan to measure the effects of having a collaborative versus a competitive type of interaction. For example, we can use a scoring system that encourages children to beat the robot and showcase more creativity in their stories.

## Data Availability

The raw data supporting the conclusions of this article will be made available by the authors, without undue reservation.

## Footnotes

1
https://ankicozmorobot.com/

2
https://beta.openai.com/

3 The dataset of story ideas with their creativity ratings used for fine-tuning the ML models is available at: https://osf.io/rm4cz/?view_only=ac999ab27fd34f6ab4de5bf1c957d92a

4
https://archive.mpi.nl/tla/elan
